# Expression and Activation of Caspase-6 in Human Fetal and Adult Tissues

**DOI:** 10.1371/journal.pone.0079313

**Published:** 2013-11-12

**Authors:** Nelly Godefroy, Bénédicte Foveau, Steffen Albrecht, Cynthia G. Goodyer, Andréa C. LeBlanc

**Affiliations:** 1 Department of Neurology and Neurosurgery, McGill University, Montreal, Quebec, Canada; 2 The Bloomfield Center for Research in Aging, Lady Davis Institute for Medical Research, Sir Mortimer B. Davis Jewish General Hospital, Montreal, Quebec, Canada; 3 Department of Pathology, McGill University, Montreal, Quebec, Canada; 4 Department of Pediatrics, McGill University Montreal, Quebec, Canada; Weizmann Institute of Science, Israel

## Abstract

Caspase-6 is an effector caspase that has not been investigated thoroughly despite the fact that Caspase-6 is strongly activated in Alzheimer disease brains. To understand the full physiological impact of Caspase-6 in humans, we investigated Caspase-6 expression. We performed western blot analyses to detect the pro-Caspase-6 and its active p20 subunit in fetal and adult lung, kidney, brain, spleen, muscle, stomach, colon, heart, liver, skin, and adrenals tissues. The levels were semi-quantitated by densitometry. The results show a ubiquitous expression of Caspase-6 in most fetal tissues with the lowest levels in the brain and the highest levels in the gastrointestinal system. Caspase-6 active p20 subunits were only detected in fetal stomach. Immunohistochemical analysis of a human fetal embryo showed active Caspase-6 positive apoptotic cells in the dorsal root ganglion, liver, lung, kidney, ovary, skeletal muscle and the intestine. In the adult tissues, the levels of Caspase-6 were lower than in fetal tissues but remained high in the colon, stomach, lung, kidney and liver. Immunohistological analyses revealed that active Caspase-6 was abundant in goblet cells and epithelial cells sloughing off the intestinal lining of the adult colon. These results suggest that Caspase-6 is likely important in most tissues during early development but is less involved in adult tissues. The low levels of Caspase-6 in fetal and adult brain indicate that increased expression as observed in Alzheimer Disease is a pathological condition. Lastly, the high levels of Caspase-6 in the gastrointestinal system indicate a potential specific function of Caspase-6 in these tissues.

## Introduction

Caspase-6 (Casp6) is one of three short pro-domain effector caspases involved in apoptotic cell death. Casp6 principally cleaves proteins containing (V/I/T/L)E(G/D)ID sites (VEIDase activity) [1]. Known Casp6 substrate proteins can be divided into two principal groups: proteins important for nuclear structure or function and intermediate filament proteins. In the nucleus, Casp6 cleaves lamin A, B and C, SP1, DNA topoisomerase I, CBP/p300, Ap-2 alpha, nuclear death domain protein p84N5, p27^KIP1^, nuclear matrix protein SATB1, emerin, phosphocholine cytidyl transferase alpha, NuMA, DFF40, and PARP [2]–[18]. The cleavage of lamin A results in the condensed chromatin of apoptotic cells [19]. In the cytosol, Casp6 cleaves desmin, vimentin and cytokeratin, proteins that are important for maintaining cellular structure and function [20]–[23].

Casp6 appears to play a major role in Alzheimer Disease (AD) pathogenesis. It has also been implicated in Huntington Disease (reviewed by [24]), in Parkinson Disease [25], and in stroke [26]. Casp6 is activated in serum-deprived human primary neurons in the absence of other effector caspases, and microinjection of active Casp6 is sufficient to induce a protracted type of cell death in primary neurons in the absence of an insult [27], [28]. Casp6 cleaves two proteins known to be involved in AD: the amyloid β precursor protein (APP) and Tau [27], [29], [30] and Casp6 activation in human primary neurons leads to increased levels of amyloid beta peptide (Aβ) [27], [31]. Casp6 also cleaves several important neuronal proteins including alpha-tubulin, and post-synaptic density proteins, Drebrin, spinophillin, actinin-1 and actinin-4 [32]. The active form of Casp6 and Tau cleaved by Casp6 (TauΔCasp6) are present in the three major neuropathological hallmarks of Alzheimer's disease: neuropil threads, neuritic plaques and neurofibrillary tangles in sporadic and familial forms of AD [33]–[35]. Casp6 is also observed in areas first affected by AD pathology in aged non-cognitively impaired and the levels of Casp6 correlated with impaired cognitive performance [34], [36]. In cultured human neurons, Casp1 activates Casp6 but it is not yet clear if Casp1 leads to Casp6 activation in AD [37]. The activity of Casp6 in the AD brains is restricted to the cytoplasm and does not localize to the neuronal nuclei as in human cerebral ischemia, whereas Casp6 is both neuritic and nuclear in morphologically apoptotic neurons [35]. Furthermore, Casp6 activity is associated with axonal degeneration in mouse sensory and human cortical primary neuron cultures [38]–[41]. Therefore, we may have a window of opportunity to inhibit Casp6 as a potential therapeutic treatment against AD.

However, the physiological function of Casp6 has not been widely investigated. Casp6 may have an important role in intestinal epithelium homeostasis [42]. Stem cells at the base of intestinal crypts migrate along the crypt-villus axis and differentiate into the specialized epithelial lining of the intestinal lumen within 3–5 days. At the luminal surface, the epithelial cells undergo anoikis, a form of apoptosis caused by the loss of cellular anchorage and resulting in the shedding of the epithelial cells into the intestinal lumen. In freshly isolated intestinal epithelial cells, detachment induces Casp6 activity before Casp3 [42]. Casp6 might be implicated during the elimination of organelles in lens development [43], [44]. However, this suggestion has been disputed since this process is not affected in Casp6 null mice [45] and the proteasome can simulate VEIDase activity [45]. Also, Casp6 activity can stimulate B lymphocyte proliferation and is implicated in B cell differentiation [46], [47].

To determine if Casp6 may play an important role in other human tissues, we examined Casp6 expression and activation in a variety of fetal and adult human tissues. The levels of Casp6 are higher in early development and decreased in most adult tissues. We find higher levels of Casp6 proenzyme in gastrointestinal tissues and active Casp6 in colonic epithelial cells of the adult. In contrast, of all tissues investigated, the brain has the lowest levels of proenzyme indicating that the high levels observed in AD are pathological. These results highlight the need to investigate the physiological role of Casp6 in peripheral tissues before inhibitors of Casp6 can be considered as a treatment for AD.

## Materials and Methods

### Source of human tissues

Fetal tissues were obtained at the time of therapeutic abortion in accordance with the ethical guidelines and regulations of the CIHR and NIH and with approval of the McGill University Institutional Review Board. Written informed consent was obtained from the mothers for the use of fetal tissues. The fetal ages ranged from 10.5–20 weeks and were calculated based on foot length [48]. Whole kidney, spleen, stomach, heart and adrenal tissues as well as samples of lung, brain, thigh muscle, colon and skin were immediately flash-frozen and stored at −80°C until processed for protein extractions. In addition, we also had a formalin-fixed paraffin embedded tissue from a fetus that was surgically removed from an ectopic tubal pregnancy; its gestational age was 13 weeks based on its crown-rump length. Informed consent was not required as surgical tissue was obtained before 1988, as established by the Quebec Civil Code. Tissue sections from one human fetal stomach of 19 weeks were obtained from Biochain® (CA, USA) for immunohistochemistry. BioChain® complies with the relevant regulations from the various governing bodies including the regulations on Good Clinical Practice (GCP) from the FDA which includes the approval of the protocol by an Investigation Review Board (IRB), informed consent form for donors, confidentiality/privacy of related information, and quality assurance in the process. Three series of −80°C frozen adult tissues were obtained from the Cooperative Human Tissue Network (CHTN, Midwestern Division, Ohio State University). CHTN-collected tissues were primarily from the same region for the stomach and kidney whereas the other tissues were not always obtained from the same area of the organ. Four frozen colon cancer and three paraffin-embedded samples with the corresponding normal tissues were also obtained from the CHTN. The CHTN policies and regulations follow current regulations and guidance for repositories from the Office of Human Research Protections (OHRP, DHHS) as defined http://www.hhs.gov/ohrp/humansubjects/guidance/ and all divisions of the CHTN operate with the review and approval of their local Institutional Review Board (IRB). The adult brain samples were obtained with written informed consent from the U. Toronto Institutional Review Board by Dr. C. Bergeron (U.Toronto), and hearts from the Cardiovascular Health Network (Institut de Cardiologie de Montréal), which were obtained under Comité universitaire d'éthique de la recherche. Seven frozen colon cancer samples, with adjacent normal tissues, were obtained by Dr. J. Faria, with ethical approval from the Jewish General Hospital Ethics Committee (Montreal, QC). All tissues were obtained under written informed consent from next-of-kin.

### Protein extraction

Tissues were homogenized in 10 volumes of ice cold RIPA buffer (150mM NaCl, 1% NP40, 0.5% sodium deoxycholate, 0.1% sodium dodecyl sulfate, 100 mM Tris pH 8, 0.1 µg/ml leupeptin, 0.2 µg/ml N-α-p-tosyl-L-lysine chloromethyl ketone hydrochloride (TLCK), 0.02 µg/ml pepstatin A, 37 µg/ml 4-(2-aminoethyl)benzenesulfonyl fluoride hydrochloride (AEBSF)) in a ground glass homogenizer. Lysates were sonicated during 5 minutes in ice and centrifuged 10 minutes at 16250 g to separate soluble and insoluble fractions. Supernatants were frozen at −80°C before use. The protein concentration for each sample was performed with BCA™ protein assay reagents (Pierce, Rockford, IL) according to the manufacturer's instructions.

### Antibodies

Monoclonal anti-Casp6_271-285_ antibody 68041A recognizes the full length and the p10 subunit of Casp6 (Pharmingen, San Diego, CA). Polyclonal anti-Casp6_16-32_ antiserum 06–691 recognizes the full length and the p20 subunit of Casp6 (Upstate, Charlottesville, VA). The anti-Casp6 10630 and 1277 are polyclonal neoepitope antisera raised against the PLDVVD C-terminal amino acids of the p20 subunit [35]. The anti-Casp1 15254 neoepitope polyclonal antiserum was raised against the PGVVWFKD C-terminal amino acids of the p20 subunit [37]. Anti-Casp1 polyclonal antiserum SC-515 was raised against the C-terminus of the p10 subunit of human Casp1 (Santa Cruz Biotechnology Inc., Santa Cruz, CA). Anti-Casp3 R280 polyclonal antisera detects the full length Casp3 (kind gift from Dr D. Nicholson, Merck Frost, Kirkland QC) and the anti-p20Casp3 antisera specific to the active Casp3 subunit was from Cell Signaling (Danvers, MA). The monoclonal anti-β-actin A5441 antibody was raised against the 16 N-terminal amino acids (Sigma, St Louis, MO).

### Western blots

One hundred micrograms of total proteins were separated by 15% sodium dodecyl sulfate-polyacrylamide gel electrophoresis, transferred to PVDF Immobilon-P membranes (Millipore, Bedford, MA), blocked in 5% milk and immunoblotted with antibodies at the following dilution: 1/250 anti-Casp6 68041A, 1/1000 anti-Casp6 06–691, 1/10000 anti-Casp6 10630, 1/1000 anti-Casp1 15254, 1/250 anti-Casp1 SC-515, 1/1000 anti-Casp3 R280, 1/1000 anti-p20Casp3, and 1/5000 anti-β-Actin A5441. Immunoreactivity was detected with 1/5000 for anti-rabbit and 1/2000 for anti-mouse horseradish peroxidase-conjugated secondary antibodies (Amersham, Oakville, ON). The β-actin immunoreactivity was detected with anti-mouse secondary immunoglobulin conjugated with alkaline phosphatase (Jackson ImmunoResearch Laboratories Inc., West Grove, PA). The blots were developed with ECL chemiluminescence (Amersham Pharmacia Biotech, Piscataway, NJ) or with 0.32 mg/ml nitro blue tetrazolium chloride, NBT and 0.16 mg/ml 5-bromo-4-chloro-3-indoyl phosphate (Promega, Madison, WI) in alkaline phosphatase buffer (100 mM Tris pH 9, 150 mM NaCl, 1 mM MgCl_2_). Gels were stained with 1.25% Coomassie blue R250 in 50% methanol and 10% acetic acid for 1 hr at room temperature and de-stained in 50% methanol and 10% acetic acid. Semi-quantitative measurement of proCasp6 levels was done with the Image Quant program on a Molecular Dynamics Personal Densitometer SI (Molecular Dynamics, Sunnyvale, CA). The volume/area values obtained from densitometric measurements were analysed using ANOVA and Dunnett's post-hoc analysis (Statview, Pickaway, NJ).

### Immunohistochemical analysis

Immunohistochemistry was performed on formalin-fixed, paraffin-embedded sections with the Ventana Benchmark Automated Immunostainer (Ventana Medical Systems, Tucson, AZ) using the company's proprietary reagent kits. After deparaffinization and rehydratation, tissue sections were treated using Ventana's Cell Conditioning Solution CC1 for 4 minutes at 100°C as described previously [35]. The primary 1277 or 10630 anti-p20Casp6 antiserum was applied at 1/2000 for 32 minutes at 42°C. Staining was detected using Ventana's diaminobenzidine detection kit according to the manufacturer's instructions. After immunostaining procedure, the sections were dehydrated, cleared in xylene mounted in Mikrokitt medium (Serum International, Montreal, Canada), and coverslipped with Tissue-Tek SCA coverslipping film (Sakura, Japan). All controls for the primary antiserum have been conducted as demonstrated in [35].

## Results

### Casp6 is ubiquitously expressed in various human fetal tissues

**Figure 1:**
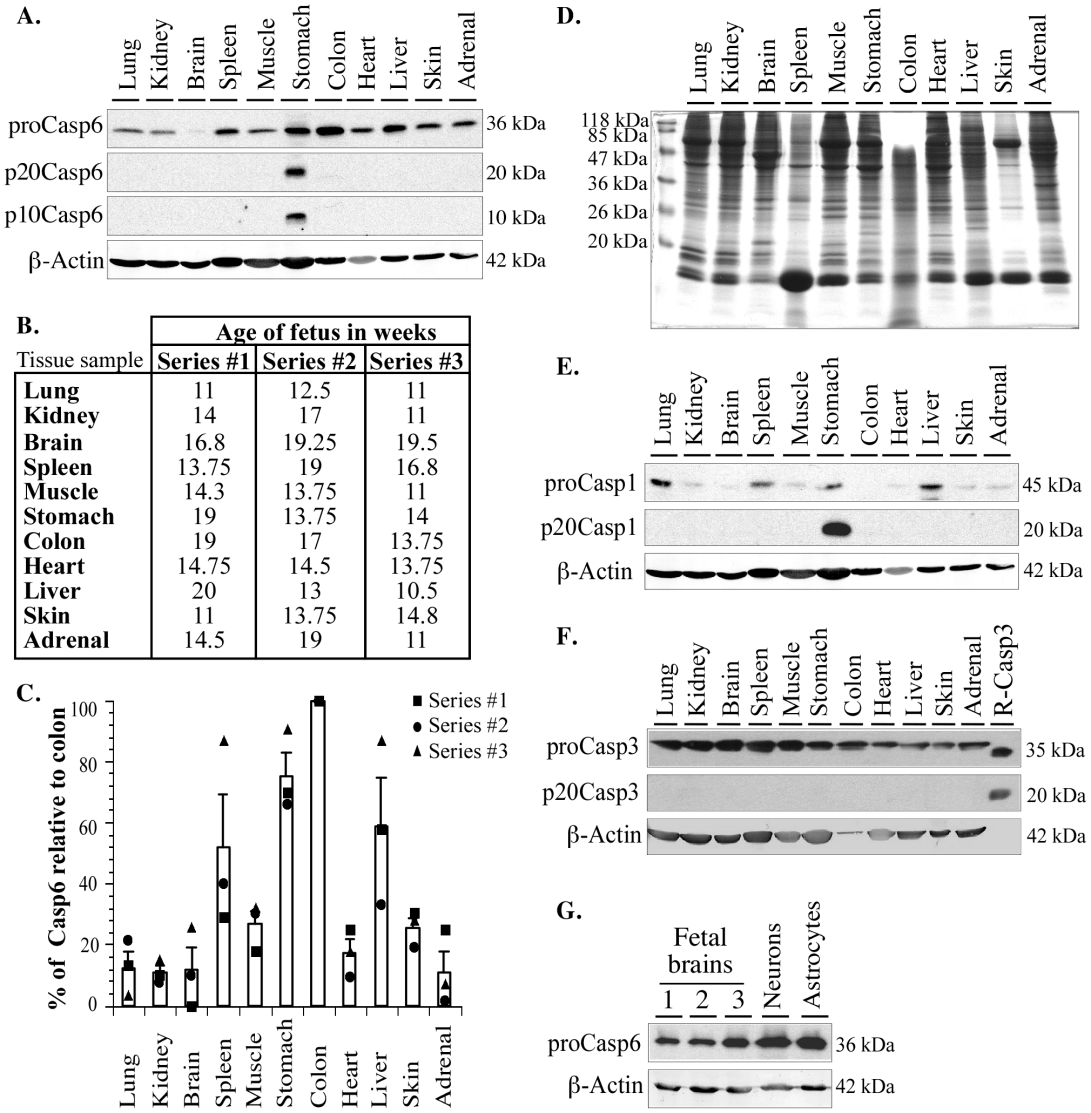
Steady state levels of Casp6, Casp1, and Casp3 in human fetal tissues. **A**. Representative western blot (series #1) of 100 µg of total proteins from fetal tissues with Upstate anti-Casp6 antisera (ProCasp6), neoepitope 10630 antisera (p20Casp6), and β-Actin antibody. **B**. Fetal age for the three series of tissues used in the study. **C**. Bar graph of proCasp6 levels relative to that in colon arbitrarily placed at 100. Data represent the individual values from series 1,2, and 3, as well as mean and SEM of 3 independent experiments. **D**. Coomassie stained gel of proteins from tissues in series 1. **E –G**. Representative western blots of 100 µg of total proteins from fetal tissues with **E**. anti-Casp1 antisera (ProCasp1), neoepitope antisera to the p20 subunit of Casp1 (p20Casp1) and β-Actin antibody, **F**. anti-Casp3 antisera (ProCasp3), neoepitope antisera to the p20 subunit of Casp3 (p20Casp3) and β-Actin antibody, and **G**. Western blot of 100 µg of total proteins from three different fetal brains, primary cultures of human fetal neurons or astrocytes with Upstate anti-Casp6 antisera (ProCasp6) or β-actin antibody.

Full-length proCasp6 is present at various levels in all 11 tissues examined (**Fig.1A**). A semi-quantitative analysis of three individual samples per tissue shows little variability in the steady state levels of proCasp6 in most tissues and there is no correlation with fetal age (**Fig. 1B & C**). The highest steady state levels of proCasp6 protein are present in colon, stomach, liver and spleen (ANOVA p<0.003). There is no significant difference in the Casp6 levels between these tissues as determined by a Dunnett's post-hoc analysis. The Casp6 levels are significantly lower (Dunnett's p<0.05) in lung, kidney, brain, heart and adrenal tissues. While it is customary to use protein markers to insure equal loading in each lane of a western blot, it is not possible to do so with varying tissues since each contains different amounts of constituent proteins like β-actin (**Fig. 1A**). However, the β-actin protein levels (**Fig. 1A**) and a Coomassie blue stain (**Fig. 1D**) insure that each lane, including the brain, lung, kidney and adrenal tissues, contains high levels of proteins and that the variability in proCasp6 protein levels amongst the various tissues is not the result of different total protein load. Casp1 levels were also examined since Casp6 can be activated by Casp1 [37]. ProCasp1 levels are highest in lung, liver, spleen and stomach and low in other tissues (**Fig. 1E**). In contrast, proCasp3 levels are high in all of the tissues examined (**Fig. 1F**).

We have previously shown that Casp6 plays an important role in stressed primary cultures of human fetal neurons and in AD. Nevertheless, the expression is weak in fetal brain samples compared to many of the tissues examined (Fig. 1A&C). We further compared proCasp6 levels in neurons and astrocytes cultured from fetal brains with those in fetal brains (Fig. 1G). Upon longer exposure than that done in Fig. 1A, proCasp6 levels are detected and are higher in the cultured cells than in the brain tissue. These results indicate that, even if the steady state levels of proCasp6 are very low compared to other tissues, there is expression of proCasp6 in the brain.

Together, these results indicate that proCasp6 and proCasp3 are expressed quite ubiquitously in human fetal tissues while proCasp1 expression is more restricted to some tissues. The high levels of proCasp6 in colon suggest a potential role for Casp6 in the physiological state of this tissue.

### Active Casp6 subunits and Casp1 p20 subunit are detected in fetal stomachs

**Figure 2:**
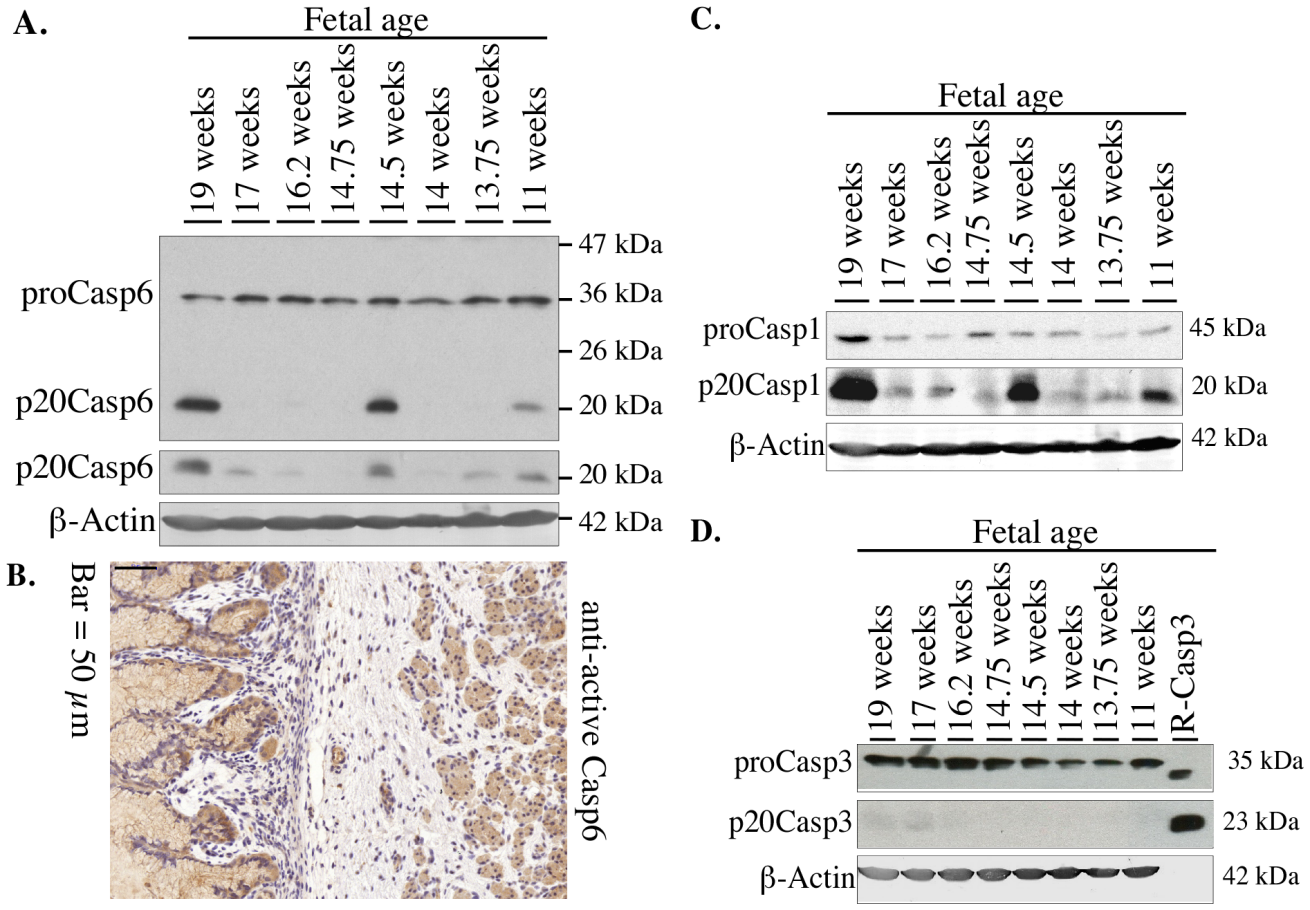
Steady state levels of Casp6, Casp1, and Casp3 in 8 different human fetal stomachs. Representative western blots containing 100 µg total protein/lane from fetal stomachs of different developmental ages with **A**. Neomarker anti-Casp6 antisera (top panel), neoepitope 10630 antisera (second panel), and β-Actin antibody (third panel), **B. Micrograph of human fetal stomach stained with 10630 anti-active Casp6 antiserum. C& D.** Western blot of (**C**) anti-Casp1 antisera (ProCasp1), neoepitope antisera to the p20 subunit of Casp1 (p20Casp1) and β-Actin antibody, and (**D**) anti-Casp3 antisera (ProCasp3), neoepitope antisera to the p20 subunit of Casp3 (p20Casp3) and β-Actin antibody.

The p10 or p20 subunits generated during the activation of Casp6 or Casp1 are detected in fetal stomach (**Fig. 1A**). However, the active Casp6 subunits were only strongly detected in the oldest of the stomach samples (19 wks), suggesting that the activation of Casp6 might be specific to the developmental stage. We therefore examined five more samples from different developmental stages ranging from 11 to 19 weeks fetal age (**Fig 2A**). While the steady state proCasp6 protein levels are similar amongst the various samples, there are considerable differences in the level of p20 subunit in the stomachs. The 19, 14.5 and 11 week samples show more p20 Casp6 subunits with the Upstate antisera. However, the more sensitive neoepitope p20Casp6 10630 antiserum detects the p20 subunit in all samples except in the 14.75 and 14 week samples. Immunostaining of fetal human stomach with the anti-active Casp6 antiserum revealed intense cytoplasmic staining of the mucosal epithelial cells (**Fig. 2B**).

Similarly, Casp1 p20 subunit is detected in fetal stomach (**Fig. 1E**). A more complete study of the stomach samples ranging from 11 to 19 weeks fetal age indicates variability in the amount of proCasp1 and p20Casp1, but with the highest levels of p20Casp1 in the same samples where p20Casp6 levels were the highest (**Fig. 2A&C**). Since Casp3 is known to activate Casp6 under certain conditions [49], we also examined the stomachs for the presence of proCasp3 and its active p17 or p20 subunit (**Fig. 2D**). Interestingly, proCasp3 levels seem to increase with development. However, there is no detection of the active p17 or p20 subunit of Casp3 in these tissues, although the antibody easily recognized recombinant Casp3 p20 subunits on the western blot. Together, the results indicate that active Casp6 subunits do not correlate with increasing stages of stomach development and that Casp1, but not Casp3, could be responsible for the activation of Casp6 in stomach.

### Active Casp6 is detected in apoptotic cells of several fetal tissues by immunohistochemistry

**Figure 3:**
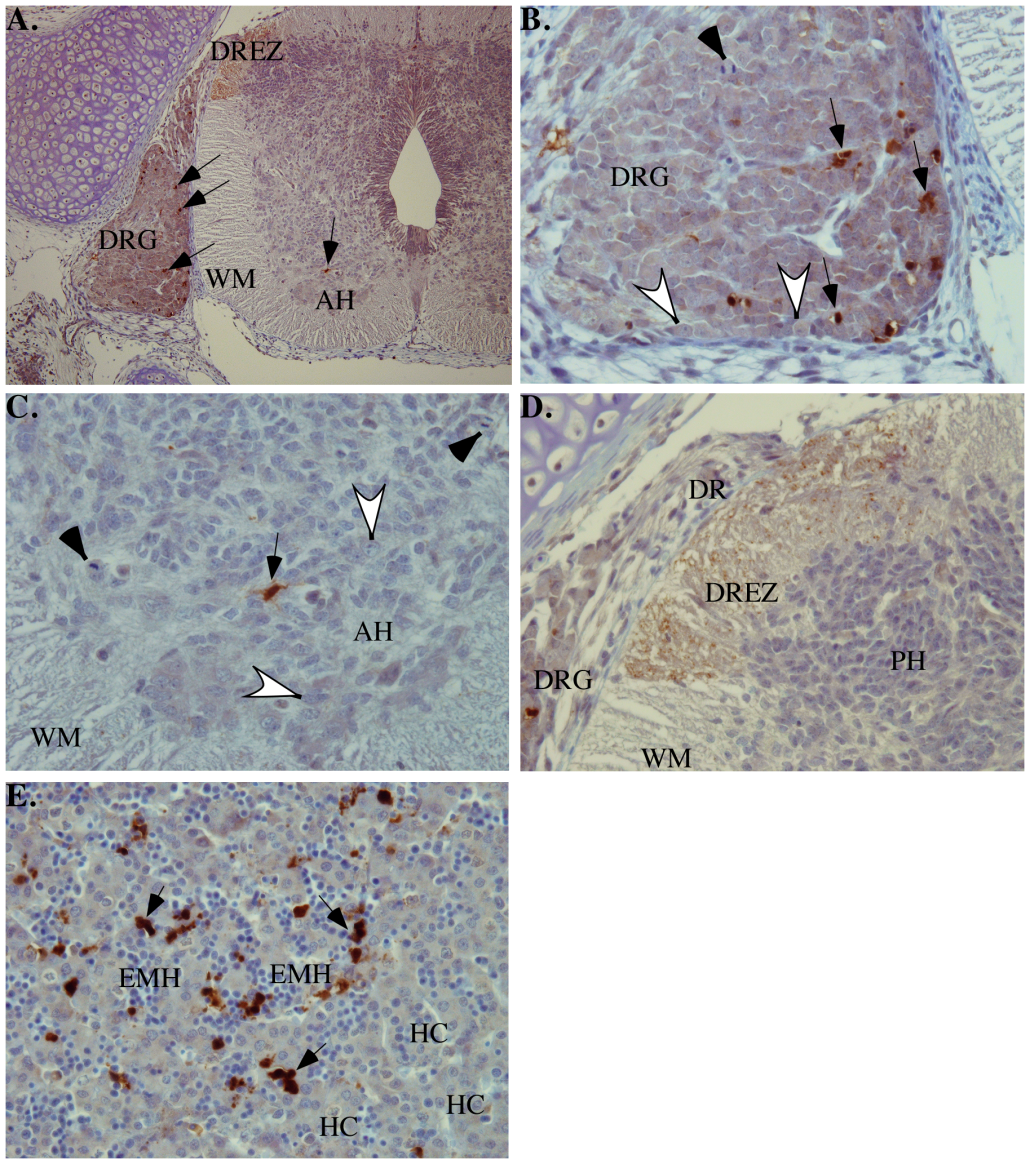
Micrographs of immunohistochemical analyses of fetal tissues with 1277 neoepitope antisera detecting the active p20 subunit of Casp6. A. At low power (original magnification, 100x), immunopositive cells (arrows) in the dorsal root ganglion (DRG), in the anterior horn (AH), and in the dorsal root entry zone (DREZ) of the spinal cord white matter (WM). B. High power view of dorsal root ganglion (DRG: original magnification, 400×) indicating mitotic activity (solid arrowhead), features of early neuronal differentiation (open arrowheads) and several apoptotic immunopositive cells (arrows). C. High power view of anterior horn (AH: original magnification, 400×) indicating dividing cells (solid arrow heads), early maturation of cells (open arrowheads), and an immunopositive apoptotic precursor cell (arrow). WM; white matter). D. High power view of dorsal spinal cord (original magnification, 400×). Immunopositive finely granular (synaptic) pattern in the dorsal root entry zone (DREZ) of the spinal cord white matter (WM). DRG, dorsal root ganglion; DR, dorsal root; PH, posterior horn). E. Extensive extramedullary hematopoiesis (EMH) in the sinusoids that separate the cords of hepatocytes (HC) in the liver (original magnification 400×). In the hematopoietic islands, there are many immunopositive apoptotic cells (arrows).

Detection of active Casp6 in tissues is only possible if there is a considerable amount of cells that have active Casp6 at any one time. Our antiserum to active Casp6 is strong and specific for immunohistochemical analyses [35], so we opted to examine a pathological sample of a fetus by immunohistochemistry to assess potential Casp6 activation in the various tissues. Cells that were immunopositive for active Casp6 were seen in many tissues. They invariably had morphological features of apoptosis, i.e., condensed cytoplasm and pyknotic fragmented nuclei. The dorsal root ganglia and the anterior horns of the spinal cord already contained cells with morphological features of early neuronal differentiation (i.e., nuclear enlargement with distinct nucleoli, increased amount of cytoplasm) and some of these were active Casp6-immunopositive (**Fig. 3A–D**). Interestingly, in the dorsal root entry zone of the spinal cord, there was also marked Casp6 immunoreactivity with a finely granular pattern (**Fig. 3A&D**). We have seen a similar pattern in fetal cerebral ischemia [35]. In the lung, there were scattered Casp6-immunoreactive cells in the stroma but not in the airway epithelium. In the liver, there were clusters of Casp6-immunoreactive cells in hematopoietic islands (**Fig. 3E** **)**; hepatocytes were not Casp6-immunoreactive. In the stomach and intestine, there were rare Casp6-immunoreactive stromal cells in the submucosa with rare Casp6-immunoreactive cells in the intestinal epithelium but none in the gastric epithelium in this case, consistent with the sporadic activation of Casp6 observed in **Fig. 2A**. In the gonad (ovary), many primary germ cells were Casp6-immunoreactive. In the kidneys, scattered stromal and tubular as well as glomerular epithelial cells were Casp6-immunoreactive. In the skeletal muscle, there were scattered Casp6-immunoreactive cells; it was not possible, based on their morphology, to determine whether these were apoptotic stromal cells or myocytes. These results indicate that Casp6 activation may be important in developmental cell death of precursor and differentiated cells of several developing tissues.

### Casp6 levels are relatively high in adult colon, stomach, spleen, kidney and lung but low in other tissues

**Figure 4:**
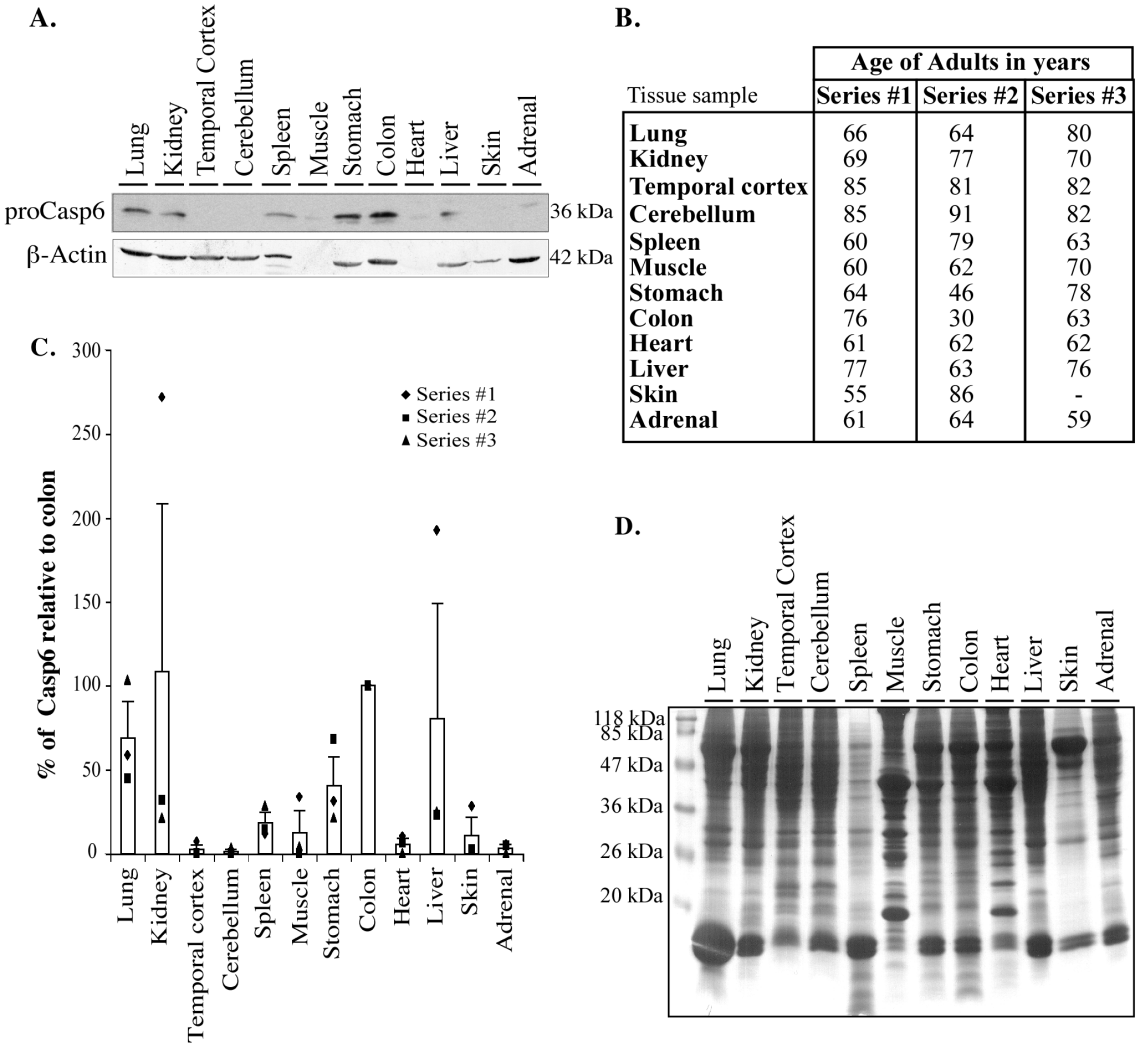
Steady state levels of Casp6, Casp1, and Casp3 in human adult tissues. **A**. Representative western blot (series #2) of 100 µg of total proteins from adult tissues with Upstate anti-Casp6 antisera (ProCasp6) and β-Actin antibody. **B**. Age of adult tissues used for the study. **C**. Bar graph of the average of proCasp6 levels relative to that in colon arbitrarily placed at 100. Data represent the individual values as well as mean and SEM of 3 independent experiments. **D**. Coomassie stained gel of proteins from tissues in series 2.

Full-length proCasp6 is detected in all 12 adult tissues examined (**Fig.4A**). The semi-quantitative analysis of the steady state levels of proCasp6 for three individual samples of variable ages (**Fig. 4B**) shows more variability than in fetal tissues (**Fig. 4C**). However, there is no correlation between the levels of proCasp6 and the age of the tissue. For example, the most variability is seen in the kidney where the ages are within 8 yrs of each other. The highest steady state levels of proCasp6 protein are in colon, lung, stomach, kidney, and liver, and the lowest levels are in spleen, muscle, skin, heart, temporal cortex, cerebellum, and adrenal tissue (ANOVA p<0.006; Dunnett's p<0.05 comparing tissues to colon). The β-actin protein levels (**Fig. 4A**) and a Coomassie blue stain (**Fig. 4D**) show that each lane contains high levels of total proteins. No β-actin was detected in muscle and heart, as expected [50] (**Fig. 4A**). In contrast to fetal tissues, there is no evidence of active Casp6 in adult tissues by western blotting (data not shown). These results indicate that the Casp6 has decreased in several adult tissues.

### The active p20 subunit of Casp6 is detected in adult colon tissue

**Figure 5:**
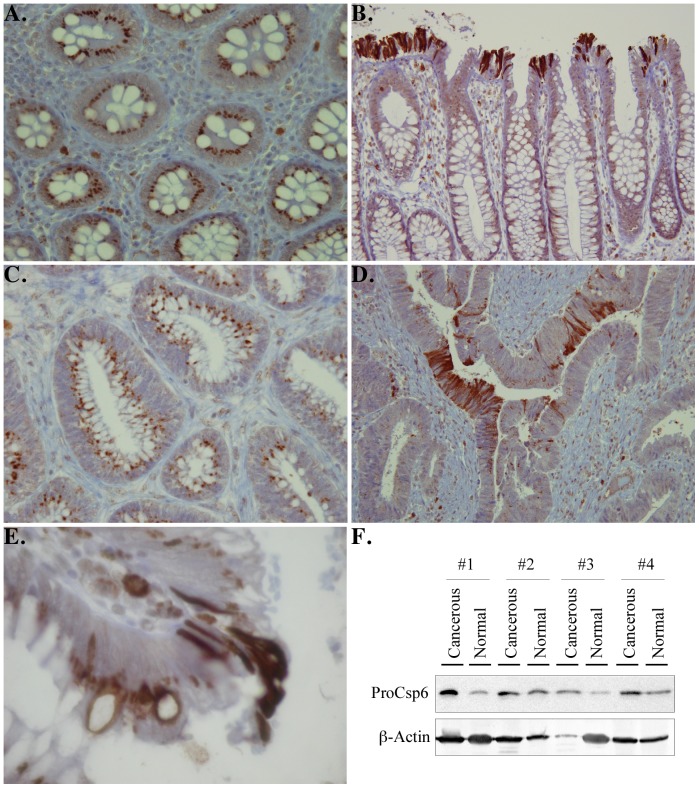
Micrographs of immunohistochemical analyses of adult normal and cancerous colon with 1277 neoepitope antisera against the active p20 subunit of Casp6. Normal colonic mucosa (A) and crypt openings (B), colonic adenocarcinomas (C) and crypt openings (D) immunostained with anti-p20Casp6 neoepitope antisera. E. Larger magnification of normal colon showing active Casp6-positive epithelial cell sloughing off in the lumen. F. Western blot of 100 µg of total proteins from normal and cancerous colon tissues with Upstate anti-Casp6 antisera (ProCasp6) and β-Actin antibody.

To assess if active Casp6 is limited to a small number of cells in adult gastrointestinal tissues and, therefore, undetectable by western blotting, we performed immunohistochemistry on normal adult colon with the anti-p20Casp6 antisera [35]. Both in normal colonic mucosa (**Fig. 5A**) and in some colonic adenocarcinomas (**Fig. 5C**), mucinous cells (goblet cells) often contain one or several cytoplasmic granules, which are immunoreactive for active Casp6. These are located between the nucleus and the mucin vacuole. In the normal colonic mucosa, mucosal cells located at the crypt openings are strongly immunoreactive for active Casp6 **(** **Fig. 5B** **)**. Cells with a similar appearance are also seen in some colonic adenocarcinomas (**Fig. 5D**). In well-differentiated areas of the cancerous tissues, one out of 10 cases has even more active Casp6 in the epithelial lining than normal tissues and one case has equivalent amounts whereas the others seem to have slightly less active Casp6 in the mucinous or epithelial cells. However, in less well-differentiated cancerous tissue, the active Casp6 is less abundant. At the crypt openings, cells that are sloughing off the epithelial lining are intensely immunostained for active Casp6 in both the cytoplasm and the nuclei (**Fig. 5E**). By western blotting, we observe that proCasp6 is in both the cancerous and normal colon tissue but there is more proCasp6 in the cancerous tissue, consistent with a decrease in the activation of Casp6 (**Fig. 5F**). Together, these results indicate that Casp6 may be highly important in the normal physiological turnover of the epithelial lining of the colon.

## Discussion

Casp6 is an effector caspase that has not been well characterized. Yet, a number of features indicate that it may play a very important role in the pathophysiology of AD. Casp6 activation increases the levels of Aβ in primary cultures of human neurons [27], [31] and in other cell types [29], [30], [51]. In addition, Casp6 cleaves tau protein, the main component of neurofibrillary tangles, and TauΔCasp6 is abundant in the neuropil threads, the neuritic plaques and the neurofibrillary tangles of sporadic and familial forms of AD [33]–[35]. Casp6 also cleaves amyloid precursor protein binding protein 1 [52]. More importantly, Casp6 activity is present in the area first affected by AD in brains from aged non-cognitively impaired individuals and the levels of Casp6 activity correlates with impaired cognitive performance in these individuals [34], [36]. The role of Casp6 in axonal degeneration [38]–[41] and not necessarily cell death [53], [54] has propelled the field to consider Casp6 as a potential novel target for AD. Here, we conducted a study to investigate the location and the level of proCasp6 in human fetal and adult tissues in order to gain more insight into the potential physiological roles of Casp6.

In contrast to what was expected, since Casp6 is highly activated in AD brains, proCasp6 levels are the lowest in normal fetal and adult brains compared to other tissues. This is consistent with results obtained by Van der Crean *et al*. who showed that mRNA levels of murine Casp6 are low in the brain [55]. Yet, we also find immunostaining evidence for active Casp6 in neurons of the dorsal root ganglia, indicating that active Casp6 is likely to participate in developmentally regulated apoptosis of the cerebrum and the peripheral nervous system. However, active Casp6 probably does not affect high numbers of neurons at any one time during normal development of human brains. Since strong activation of Casp6 occurs in AD and ischemic human brains [35], the low levels of proCasp6 may assure long term survival of the neurons after developmental cell death through Casp3 activity-dependent pruning [56] to generate a functional neuritic network.

Interestingly, proCasp6 is highly expressed in most fetal tissues and its levels decrease in many of the adult tissues. Furthermore, there is little variability in the levels of proCasp6 amongst the different fetal samples of one tissue. These results indicate that Casp6 may play an important role in the fetal tissues. Whether this role is in the regulation of apoptosis or in another yet undefined function of Casp6 is not known. Effector caspases can perform non-apoptotic functions in some tissues. For example, Casp3 regulates muscle cell differentiation [57]. Casp6 may be important in organelle dissolution in the lens of the eye [43]-[45] and is involved in the proliferation and differentiation of B lymphocytes [46], [47]. The immunohistochemical staining of tissues shows that the active Casp6 is associated with apoptotic morphology in several tissues. However, there was also a fine granular pattern of staining reminiscent of synaptic staining in the dorsal root entry zone, which could indicate a role for Casp6 in remodeling of synapses. Therefore, although it is likely that active Casp6 induces cell death in the developmental maturation of a number of tissues, it may have other functions.

In fetal stomach, we find active Casp6 p20 subunits in several samples indicating that the activity of Casp6 is involved in fetal stomach development. There is variability in the level of active Casp6 in the different stomach samples taken from different stages of development but the anti-p20Casp6 antiserum detects active Casp6 in most of the samples analysed. The size and development of the human fetal stomach depends on the size of the fetus and the amount of swallowed amniotic fluid [58]. Therefore, the developmental stage in weeks may not be directly comparable from one fetus to the other. The differentiation of epithelial cells occurs between 10 and 20 weeks of fetal development in humans, the stage of development studied herein [59], suggesting that active Casp6 may be involved in the remodeling of the intestine epithelial lining during development. Interestingly, the activation of Casp6 is accompanied by active Casp1, a caspase known to regulate activation of Casp6 in human neurons [37]. Furthermore, the loss of Casp1 expression has been observed in 19.3% of gastric carcinomas [60]. While we detect initiator Casp1 active subunits in the fetal stomach where Casp6 was also activated, we fail to see the active subunits of Casp3 in this tissue. This does not indicate clearly the absence of Casp3 activity because the absence of caspase subunits in tissues may be the result of high turnover, rapid clearance of the apoptotic cell by macrophages or simply low number of cells with the active caspase subunit in the tissues. However, we can conclude that, in the stomach, Casp6 could be activated through Casp1. In the normal adult colon, proCasp6 levels are also quite high and the active Casp6 is very abundant in colonic mucosa and epithelial cell anoikis. This result is consistent with the rapid activation of Casp6 in mechanically detached gastrointestinal epithelial cell primary cultures [42]. Together, these results suggest an important physiological function for Casp6 in both the developing and adult gastrointestinal system.

Colorectal cancer is believed to result from an inhibition of apoptosis [61]. Similar to normal colon, well differentiated colonic mucosa of adenocarcinomas are also immunostained with anti-p20Casp6. However, in less well-differentiated areas, active Casp6 is strongly decreased consistent with the higher levels of proCasp6 in cancer tissues that indicate less activation of Casp6. It is unlikely that the increased levels of Casp6 are due to p53 transactivation since all except one tissue were strongly positive for non-functional p53 (data not shown) [62]. Recently, it has been reported that resveratrol induces Casp6-dependent apoptosis in colon cancer cell lines, suggesting that apoptosis in colon carcinoma cells can be induced in the absence of other pro-apoptotic proteins like Bax or p53 [63]. Therefore, the loss of Casp6 activation may significantly contribute to the cancer state in colon.

In conclusion, we have shown that the Casp6 most likely plays an important physiological role in gastrointestinal tissues and that Casp6 may have important functions in the development of many fetal tissues. We have further shown the loss of active Casp6 in less well-differentiated colon cancer tissues. Therefore, while inhibition of the Casp6 activation in AD brains can be considered as a potential therapy to prevent further pathophysiology, these results indicate that the therapeutic approach will need to carefully consider potential side effects on the gastrointestinal system.

## References

[1] Thornberry NA, Rano TA, Peterson EP, Rasper DM, Timkey T, et al (1997) A combinatorial approach defines specificities of members of the caspase family and granzyme B. Functional relationships established for key mediators of apoptosis. J Biol Chem 272: 17907–17911. 9218414 10.1074/jbc.272.29.17907

[2] Rickers A, Peters N, Badock V, Beyaert R, Vandenabeele P, et al (1999) Cleavage of transcription factor SP1 by caspases during anti-IgM-induced B-cell apoptosis. Eur J Biochem 261: 269–274. 10103059 10.1046/j.1432-1327.1999.00273.x

[3] Samejima K, Svingen PA, Basi GS, Kottke T, Mesner PW Jr, et al (1999) Caspase-mediated cleavage of DNA topoisomerase I at unconventional sites during apoptosis. J Biol Chem 274: 4335–4340. 9933635 10.1074/jbc.274.7.4335

[4] Rouaux C, Jokic N, Mbebi C, Boutillier S, Loeffler JP, et al (2003) Critical loss of CBP/p300 histone acetylase activity by caspase-6 during neurodegeneration. EMBO J 22: 6537–6549. 14657026 10.1093/emboj/cdg615 PMC291810

[5] Nyormoi O, Wang Z, Doan D, Ruiz M, McConkey D, et al (2001) Transcription factor AP-2alpha is preferentially cleaved by caspase 6 and degraded by proteasome during tumor necrosis factor alpha-induced apoptosis in breast cancer cells. Mol Cell Biol 21: 4856–4867. 11438643 10.1128/MCB.21.15.4856-4867.2001 PMC87191

[6] Liu X, Zou H, Widlak P, Garrard W, Wang X (1999) Activation of the apoptotic endonuclease DFF40 (caspase-activated DNase or nuclease). Oligomerization and direct interaction with histone H1. J Biol Chem 274: 13836–13840. 10318789 10.1074/jbc.274.20.13836

[7] Buendia B, Santa-Maria A, Courvalin JC (1999) Caspase-dependent proteolysis of integral and peripheral proteins of nuclear membranes and nuclear pore complex proteins during apoptosis. J Cell Sci 112: 1743–1753. 10318766 10.1242/jcs.112.11.1743

[8] Doostzadeh-Cizeron J, Yin S, Goodrich DW (2000) Apoptosis induced by the nuclear death domain protein p84N5 is associated with caspase-6 and NF-kappa B activation. J Biol Chem 275: 25336–25341. 10840029 10.1074/jbc.M000793200

[9] Cross T, Griffiths G, Deacon E, Sallis R, Gough M, et al (2000) PKC-delta is an apoptotic lamin kinase. Oncogene 19: 2331–2337. 10822384 10.1038/sj.onc.1203555

[10] Chiarini A, Whitfield JF, Armato U, Dal Pra I (2002) Protein kinase C-beta II Is an apoptotic lamin kinase in polyomavirus-transformed, etoposide-treated pyF111 rat fibroblasts. J Biol Chem 277: 18827–18839. 11901153 10.1074/jbc.M111921200

[11] Eymin B, Sordet O, Droin N, Munsch B, Haugg M, et al (1999) Caspase-induced proteolysis of the cyclin-dependent kinase inhibitor p27Kip1 mediates its anti-apoptotic activity. Oncogene 18: 4839–4847. 10490817 10.1038/sj.onc.1202860

[12] Columbaro M, Mattioli E, Lattanzi G, Rutigliano C, Ognibene A, et al (2001) Staurosporine treatment and serum starvation promote the cleavage of emerin in cultured mouse myoblasts: involvement of a caspase-dependent mechanism. FEBS Lett 509: 423–429. 11749967 10.1016/s0014-5793(01)03203-3

[13] Galande S, Dickinson LA, Mian IS, Sikorska M, Kohwi-Shigematsu T (2001) SATB1 cleavage by caspase 6 disrupts PDZ domain-mediated dimerization, causing detachment from chromatin early in T-cell apoptosis. Mol Cell Biol 21: 5591–5604. 11463840 10.1128/MCB.21.16.5591-5604.2001 PMC87280

[14] Gotzmann J, Meissner M, Gerner C (2000) The fate of the nuclear matrix-associated-region-binding protein SATB1 during apoptosis. Cell Death Differ 7: 425–438. 10800076 10.1038/sj.cdd.4400668

[15] Hirata H, Takahashi A, Kobayashi S, Yonehara S, Sawai H, et al (1998) Caspases are activated in a branched protease cascade and control distinct downstream processes in Fas-induced apoptosis. J Exp Med 187: 587–600. 9463409 10.1084/jem.187.4.587 PMC2212161

[16] Lagace TA, Miller JR, Ridgway ND (2002) Caspase processing and nuclear export of CTP:phosphocholine cytidylyltransferase alpha during farnesol-induced apoptosis. Mol Cell Biol 22: 4851–4862. 12052891 10.1128/MCB.22.13.4851-4862.2002 PMC133913

[17] Shimizu T, Cao CX, Shao RG, Pommier Y (1998) Lamin B phosphorylation by protein kinase calpha and proteolysis during apoptosis in human leukemia HL60 cells. J Biol Chem 273: 8669–8674. 9535842 10.1074/jbc.273.15.8669

[18] Slee EA, Adrain C, Martin SJ (2001) Executioner caspase-3, -6, and -7 perform distinct, non-redundant roles during the demolition phase of apoptosis. J Biol Chem 276: 7320–7326. 11058599 10.1074/jbc.M008363200

[19] Ruchaud S, Korfali N, Villa P, Kottke TJ, Dingwall C, et al (2002) Caspase-6 gene disruption reveals a requirement for lamin A cleavage in apoptotic chromatin condensation. EMBO J 21: 1967–1977. 11953316 10.1093/emboj/21.8.1967 PMC125972

[20] Caulin C, Salvesen GS, Oshima RG (1997) Caspase cleavage of keratin 18 and reorganization of intermediate filaments during epithelial cell apoptosis. J Cell Biol 138: 1379–1394. 9298992 10.1083/jcb.138.6.1379 PMC2132555

[21] Byun Y, Chen F, Chang R, Trivedi M, Green KJ, et al (2001) Caspase cleavage of vimentin disrupts intermediate filaments and promotes apoptosis. Cell Death Differ 8: 443–450. 11423904 10.1038/sj.cdd.4400840

[22] Prasad S, Soldatenkov VA, Srinivasarao G, Dritschilo A (1999) Intermediate filament proteins during carcinogenesis and apoptosis (Review). Int J Oncol 14: 563–570. 10024692 10.3892/ijo.14.3.563

[23] Chen F, Chang R, Trivedi M, Capetanaki Y, Cryns VL (2003) Caspase proteolysis of desmin produces a dominant-negative inhibitor of intermediate filaments and promotes apoptosis. J Biol Chem 278: 6848–6853. 12477713 10.1074/jbc.M212021200

[24] Graham RK, Ehrnhoefer DE, Hayden MR (2011) Caspase-6 and neurodegeneration. Trends Neurosci 34: 646–656. 22018804 10.1016/j.tins.2011.09.001

[25] Giaime E, Sunyach C, Druon C, Scarzello S, Robert G, et al (2010) Loss of function of DJ-1 triggered by Parkinson's disease-associated mutation is due to proteolytic resistance to caspase-6. Cell Death Differ 17: 158–169. 19680261 10.1038/cdd.2009.116 PMC2796338

[26] Akpan N, Serrano-Saiz E, Zacharia BE, Otten ML, Ducruet AF, et al (2011) Intranasal delivery of caspase-9 inhibitor reduces caspase-6-dependent axon/neuron loss and improves neurological function after stroke. J Neurosci 31: 8894–8904. 21677173 10.1523/JNEUROSCI.0698-11.2011 PMC3143191

[27] LeBlanc AC, Liu H, Goodyer C, Bergeron C, Hammond J (1999) Caspase-6 role in apoptosis of human neurons, amyloidogenesis and Alzheimer's Disease. J Biol Chem 274: 23426–23436. 10438520 10.1074/jbc.274.33.23426

[28] Zhang Y, Goodyer C, LeBlanc A (2000) Selective and protracted apoptosis in human primary neurons microinjected with active caspase-3, -6, -7, and -8. J Neurosci 20: 8384–8389. 11069945 10.1523/JNEUROSCI.20-22-08384.2000 PMC6773170

[29] Weidemann A, Paliga K, Durrwang U, Reinhard FB, Schuckert O, et al (1999) Proteolytic processing of the Alzheimer's disease amyloid precursor protein within its cytoplasmic domain by caspase-like proteases. J Biol Chem 274: 5823–5829. 10026204 10.1074/jbc.274.9.5823

[30] Pellegrini L, Passer B, Tabaton M, Ganjei K, D'Adamio L (1999) Alternative, non-secretase processing of Alzheimer's β-amyloid precursor protein during apoptosis by caspase-6 and -8. J Biol Chem 274: 21011–21016. 10409650 10.1074/jbc.274.30.21011

[31] LeBlanc A (1995) Increased production of 4 kDa amyloid beta peptide in serum deprived human primary neuron cultures: possible involvement of apoptosis. J Neurosci 15: 7837–7846. 8613723 10.1523/JNEUROSCI.15-12-07837.1995 PMC6577962

[32] Klaiman G, Petzke TL, Hammond J, LeBlanc AC (2008) Targets of caspase-6 activity in human neurons and Alzheimer disease. Mol Cell Proteomics 7: 1541–1555. 18487604 10.1074/mcp.M800007-MCP200 PMC2500235

[33] Albrecht S, Bogdanovic N, Ghetti B, Winblad B, LeBlanc AC (2009) Caspase-6 activation in familial Alzheimer disease brains carrying amyloid precursor protein or presenilin I or presenilin II mutations. J Neuropathol Exp Neurol 68: 1282–1293. 19915487 10.1097/NEN.0b013e3181c1da10 PMC3079356

[34] Albrecht S, Bourdeau M, Bennett D, Mufson EJ, Bhattacharjee M, et al (2007) Activation of caspase-6 in aging and mild cognitive impairment. Am J Pathol 170: 1200–1209. 17392160 10.2353/ajpath.2007.060974 PMC1829454

[35] Guo H, Albrecht S, Bourdeau M, Petzke T, Bergeron C, et al (2004) Active Caspase-6 and Caspase-6 cleaved Tau in neuropil threads, neuritic plaques and neurofibrillary tangles of Alzheimer's Disease. Am J Pathol 165: 523–531. 15277226 10.1016/S0002-9440(10)63317-2 PMC1618555

[36] Ramcharitar J, V.M A, Albrecht S, Bennett DA, LeBlanc AC (2013) Caspase-6 activity predicts lower episodic memory ability in aged individuals. Neurobiol Aging 34: 1815–1824. 23402898 10.1016/j.neurobiolaging.2013.01.007 PMC3772349

[37] Guo H, Petrin D, Zhang Y, Bergeron C, Goodyer CG, et al (2006) Caspase-1 activation of caspase-6 in human apoptotic neurons. Cell Death Differ 13: 285–292. 16123779 10.1038/sj.cdd.4401753

[38] Lee SC, Chan JY, Pervaiz S (2010) Spontaneous and 5-fluorouracil-induced centrosome amplification lowers the threshold to resveratrol-evoked apoptosis in colon cancer cells. Cancer Lett 288: 36–41. 19616374 10.1016/j.canlet.2009.06.020

[39] Simon DJ, Weimer RM, McLaughlin T, Kallop D, Stanger K, et al (2012) A caspase cascade regulating developmental axon degeneration. J Neurosci 32: 17540–17553. 23223278 10.1523/JNEUROSCI.3012-12.2012 PMC3532512

[40] Sivananthan S, Lee A, Goodyer CG, LeBlanc AC (2010) Familial amyloid precursor protein mutants cause caspase-6-dependent but amyloid β-peptide-independent neuronal degeneration in primary human neuron cultures. Cell Death Dis 1: .e100. 10.1038/cddis.2010.74 PMC3032318 21368865

[41] Uribe V, Wong BK, Graham RK, Cusack CL, Skotte NH, et al (2012) Rescue from excitotoxicity and axonal degeneration accompanied by age-dependent behavioral and neuroanatomical alterations in caspase-6-deficient mice. Hum Mol Genet 21: 1954–1967. 22262731 10.1093/hmg/dds005 PMC3315204

[42] Grossmann J, Mohr S, Lapentina EG, Fiocchi C, Levine AD (1998) Sequential and rapid activation of select caspases during apoptosis of normal intestinal epithelial cells. Am J Physiol 274: G1117–1124. 9696713 10.1152/ajpgi.1998.274.6.G1117

[43] Morozov V, Wawrousek EF (2006) Caspase-dependent secondary lens fiber cell disintegration in {alpha}A-/{alpha}B-crystallin double-knockout mice. Development 133: 813–821. 16439475 10.1242/dev.02262

[44] Foley JD, Rosenbaum H, Griep AE (2004) Temporal regulation of VEID-7-amino-4-trifluoromethylcoumarin cleavage activity and caspase-6 correlates with organelle loss during lens development. J Biol Chem 279: 32142–32150. 15161922 10.1074/jbc.M313683200

[45] Zandy AJ, Lakhani S, Zheng T, Flavell RA, Bassnett S (2005) Role of the executioner caspases during lens development. J Biol Chem 280: 30263–30272. 15994297 10.1074/jbc.M504007200

[46] Olson NE, Graves JD, Shu GL, Ryan EJ, Clark EA (2003) Caspase activity is required for stimulated B lymphocytes to enter the cell cycle. J Immunol 170: 6065–6072. 12794135 10.4049/jimmunol.170.12.6065

[47] Watanabe C, Shu GL, Zheng TS, Flavell RA, Clark EA (2008) Caspase 6 regulates B cell activation and differentiation into plasma cells. J Immunol 181: 6810–6819. 18981099 10.4049/jimmunol.181.10.6810 PMC2728076

[48] Munsick RA (1984) Human foetal extremity lengths in the interval from 9 to 21 menstrual weeks of pregnancy. Am J Obstet Gynecol 149: 883–887. 6465253 10.1016/0002-9378(84)90609-4

[49] Chan JY, Phoo MS, Clement MV, Pervaiz S, Lee SC (2008) Resveratrol displays converse dose-related effects on 5-fluorouracil-evoked colon cancer cell apoptosis: the roles of caspase-6 and p53. Cancer Biol Ther 7: 1305–1312. 18497562 10.4161/cbt.7.8.6302

[50] Ilkovski B, Clement S, Sewry C, North KN, Cooper ST (2005) Defining alpha-skeletal and alpha-cardiac actin expression in human heart and skeletal muscle explains the absence of cardiac involvement in ACTA1 nemaline myopathy. Neuromuscul Disord 15: 829–835. 16288873 10.1016/j.nmd.2005.08.004

[51] Gervais F, Xu D, Robertson G, Vaillancourt J, Zhu Y, et al (1999) Involvement of caspases in proteolytic cleavage of Alzheimer's β-amyloid precursor protein and amyloidogenic β-peptide formation. Cell 97: 395–406. 10319819 10.1016/s0092-8674(00)80748-5

[52] Chen Y, McPhie DL, Hirschberg J, Neve RL (2000) The amyloid precursor protein-binding protein APP-BP1 drives the cell cycle through the S-M checkpoint and causes apoptosis in neurons. J Biol Chem 275: 8929–8935. 10722740 10.1074/jbc.275.12.8929

[53] Gray DC, Mahrus S, Wells JA (2010) Activation of specific apoptotic caspases with an engineered small-molecule-activated protease. Cell 142: 637–646. 20723762 10.1016/j.cell.2010.07.014 PMC3689538

[54] Klaiman G, Champagne N, LeBlanc AC (2009) Self-activation of Caspase-6 in vitro and in vivo: Caspase-6 activation does not induce cell death in HEK293T cells. Biochim Biophys Acta 1793: 592–601. 19133298 10.1016/j.bbamcr.2008.12.004

[55] Van de Craen M, Vandenabeele P, Declercq W, Van den Brande I, Van Loo G, et al (1997) Characterization of seven murine caspase family members. FEBS Lett 403: 61–69. 9038361 10.1016/s0014-5793(97)00026-4

[56] Kuida K, Zheng TS, Na S, Kuan C, Yang D, et al (1996) Decreased apoptosis in the brain and premature lethality in CPP32-deficient mice. Nature 384: 368–372. 8934524 10.1038/384368a0

[57] Dee K, Freer M, Mei Y, Weyman CM (2002) Apoptosis coincident with the differentiation of skeletal myoblasts is delayed by caspase 3 inhibition and abrogated by MEK-independent constitutive Ras signaling. Cell Death Differ 9: 209–218. 11840171 10.1038/sj.cdd.4400930

[58] Cetin E, Malas MA, Albay S, Cankara N (2006) The development of stomach during the fetal period. Surg Radiol Anat 28: 438–446. 16906359 10.1007/s00276-006-0124-x

[59] Chailler P, Menard D (1999) Ontogeny of EGF receptors in the human gut. Front Biosci 4: D87–101. 9889180 10.2741/chailler

[60] Jee CD, Lee HS, Bae SI, Yang HK, Lee YM, et al (2005) Loss of caspase-1 gene expression in human gastric carcinomas and cell lines. Int J Oncol 26: 1265–1271. 15809717

[61] Bedi A, Pasricha PJ, Akhtar AJ, Barber JP, Bedi GC, et al (1995) Inhibition of apoptosis during development of colorectal cancer. Cancer Res 55: 1811–1816. 7728743

[62] MacLachlan TK, El-Deiry WS (2002) Apoptotic threshold is lowered by p53 transactivation of caspase-6. Proc Natl Acad Sci U S A 99: 9492–9497. 12089322 10.1073/pnas.132241599 PMC123168

[63] Lee SC, Chan J, Clement MV, Pervaiz S (2006) Functional proteomics of resveratrol-induced colon cancer cell apoptosis: caspase-6-mediated cleavage of lamin A is a major signaling loop. Proteomics 6: 2386–2394. 16518869 10.1002/pmic.200500366

